# The Safety and Tolerability of 5-Aminolevulinic Acid Phosphate with Sodium Ferrous Citrate in Patients with Type 2 Diabetes Mellitus in Bahrain

**DOI:** 10.1155/2016/8294805

**Published:** 2016-09-22

**Authors:** Feryal Al-Saber, Waleed Aldosari, Mariam Alselaiti, Hesham Khalfan, Ahmed Kaladari, Ghulam Khan, George Harb, Riyadh Rehani, Sizuka Kudo, Aya Koda, Tohru Tanaka, Motowo Nakajima, Abdulla Darwish

**Affiliations:** ^1^Bahrain Defense Force Hospital/Royal Medical Services, Riffa, Bahrain; ^2^SBI Pharmaceuticals Middle East and North Africa, Seef, Bahrain; ^3^DZS Clinical Services, Bound Brook, NJ, USA; ^4^SBI Pharmaceuticals, Tokyo, Japan

## Abstract

Type 2 diabetes mellitus is prevalent especially in Gulf countries and poses serious long-term risks to patients. A multifaceted treatment approach can include nutritional supplements with antioxidant properties such as 5-aminolevulinic acid (5-ALA) with sodium ferrous citrate (SFC). This prospective, randomized, single-blind, placebo-controlled, dose escalating pilot clinical trial assessed the safety of 5-ALA with SFC at doses up to 200 mg 5-ALA/229.42 mg SFC per day in patients living in Bahrain with type 2 diabetes mellitus that was uncontrolled despite the use of one or more antidiabetic drugs. Fifty-three patients (*n* = 53) from 3 sites at one center were enrolled by Dr. Feryal (Site #01), Dr. Hesham (Site #02), and Dr. Waleed (Site #03) (*n* = 35, 5-ALA-SFC; *n* = 18, placebo). There was no significant difference in incidence of adverse events reported, and the most frequent events reported were gastrointestinal in nature, consistent with the known safety profile of 5-ALA in patients with diabetes. No significant changes in laboratory values and no difference in hypoglycemia between patients receiving 5-ALA and placebo were noted. Overall, the current results support that use of 5-ALA-SFC up to 200 mg per day taken as 2 divided doses is safe in patients taking concomitant oral antidiabetic medications and may offer benefits in the diabetic population. This trial is registered with ClinicalTrials.gov NCT02481141.

## 1. Introduction

Type 2 diabetes mellitus is a prevalent chronic disease, which consumes considerable medical resources and has serious long-term clinical consequences. It is currently estimated that 415 million people worldwide have diabetes mellitus [1]. Diabetes is of particular concern in Bahrain, with local prevalence of 15.6% [1] as compared with worldwide prevalence of approximately 9% [2]. Obesity is a major risk factor for type 2 diabetes, and in some Gulf countries obesity is reaching epidemic proportions [3]. Visceral fat secretes inflammatory markers, and subclinical inflammation has been linked to oxidative stress in adipose tissue, which may play a part in insulin resistance and the development and progression of type 2 diabetes mellitus [3]. Type 2 diabetes is a progressive disease with pancreatic *β*-cell function declining over time, likely involving oxidative stress from increased generation of reactive oxygen species (ROS). Oxidative stress may also play a role in the pathogenesis of microvascular complications of diabetes [4].

Treatment for type 2 diabetes mellitus consists of a multifaceted approach implemented in a stepwise fashion, beginning with lifestyle modifications such as improved diet and exercise as the first-line treatment and progressing to oral antidiabetic agents [5]. Dietary antioxidants may be of particular interest in this population, and animal studies support that adequate dietary antioxidants may alleviate complications of diabetes by protecting against oxidative stress [4]. A range of over-the-counter nutraceutical products are available that may be beneficial in the population with diabetes and metabolic syndrome, and research has shown a high level of use of dietary supplements in the population [6]. Given the high use of nutritional supplements in patients with diabetes and concomitant use of antidiabetic medications, it is important to assess potential side effects and safety, including drug interactions of dietary supplements [4].

5-Aminolevulinic acid (5-ALA) is a natural delta amino acid widely present in nature that plays an important role in living organisms. Trace amounts of 5-ALA exist in common foods such as bananas, spinach, tomatoes, and mushrooms, and larger amounts are found in fermented products such as wine [7]. 5-ALA and iron are critical components in the body's synthesis of heme, a major component of hemoglobin and myoglobin and also an essential component of cytochromes and therefore a critical component of the energy generating function of mitochondria. Mitochondrial dysfunction has been associated with insulin resistance and type 2 diabetes mellitus [8], and research has shown that increased glucose production leads to decreased 5-ALA production [9]. Preclinical and clinical studies indicate that dietary supplementation with 5-ALA, which has antioxidant properties, combined with SFC could be beneficial in patients with diabetes [7, 9–11].

Currently, 5-ALA phosphate is approved as a nutritional supplement in Japan, and 5-ALA phosphate in combination with sodium ferrous citrate (SFC) has been approved as a nutritional supplement and marketed in Japan, Philippines, Dubai, Vietnam, Jordan, and Bahrain. 5-ALA hydrochloride is approved in 39 countries and indicated for intraoperative resection of glioma under fluorescence guidance. Clinical studies have shown improved glucose metabolism after 12-week administration of 5-ALA plus sodium ferrous citrate (SFC) as measured by a 2-hour oral glucose tolerance test [9, 10] and support the safety of doses of 50 mg 5-ALA and 57.4 mg SFC in patients with type 2 diabetes mellitus treated with oral antidiabetic agents [7].

The objective of the current study was to investigate the safety and preliminary efficacy of doses up to 200 mg 5-ALA-SFC in a population of patients with type 2 diabetes mellitus living in Bahrain.

## 2. Materials and Methods

### 2.1. Study Design

This study was a prospective, randomized, single-blind, placebo-controlled, dose escalating pilot clinical trial. The study protocol was approved by the Research Ethics Committee of the Bahrain Defense Force Royal Medical Services and the National Health Regulatory Agency (NHRA) in the Kingdom of Bahrain and was registered to ClinicalTrials.gov as NCT02481141.

Eligible patients were males and females residing in Bahrain, between 20 and 75 years old, who gave written informed consent, were diagnosed with type 2 diabetes mellitus with HbA1c ≥6.5 and ≤10% that was uncontrolled despite the use of one or more antidiabetic drugs, had BMI ≤  44 kg/m^2^, sitting BP ≤ 160/100 mmHg, negative sleep apnea screen, and ophthalmological exam within normal limits as judged by the investigator, and otherwise were considered in good health in the opinion of the investigator based on results of medical history, physical exam, and clinical laboratory assessments. Female patients were not pregnant and not breast-feeding and, if of childbearing potential, agreed to use an acceptable method of birth control during the study. Patients were ineligible if they had hepatic dysfunction defined as liver function tests ≥1.5 times the upper limit of normal, renal dysfunction defined as BUN and/or serum creatinine ≥1.5 times the upper limit of normal and/or eGFR <30 mL/min/1.73 m^2^, history of any life-threatening disease, cardiovascular disease, viral hepatitis, porphyria, or hemochromatosis. Patients also were not enrolled if they had an allergy to ALA, SFC, or any other component of study product, used insulin for management of diabetes, had a hypoglycemic event within the previous 3 months defined as serum glucose levels less than 70 mg/dL, or had a history of sickle cell anemia disease.

Patients were enrolled from 3 clinics at one investigational site at the Bahrain Defense Force Hospital in Manama, Bahrain, from May 2014 to July 2015. After informed consent was obtained, screening procedures were performed including a medical history, physical exam, 12-lead ECG, laboratory assessment, ophthalmologic exam, and screening for sleep apnea. Glucometers and necessary supplies were provided at screening for daily monitoring of glucose levels, and patients were required to record daily glucose levels prior to breakfast and 2 hours after breakfast for at least 7 consecutive days during a run-in phase prior to week 0 randomization visit. Patients continued to monitor daily glucose levels for study purposes prior to breakfast and 2 hours after breakfast until week 12's visit. These daily measurements were recorded electronically and were downloaded from a flash drive by the site at each visit.

**Figure 1:**
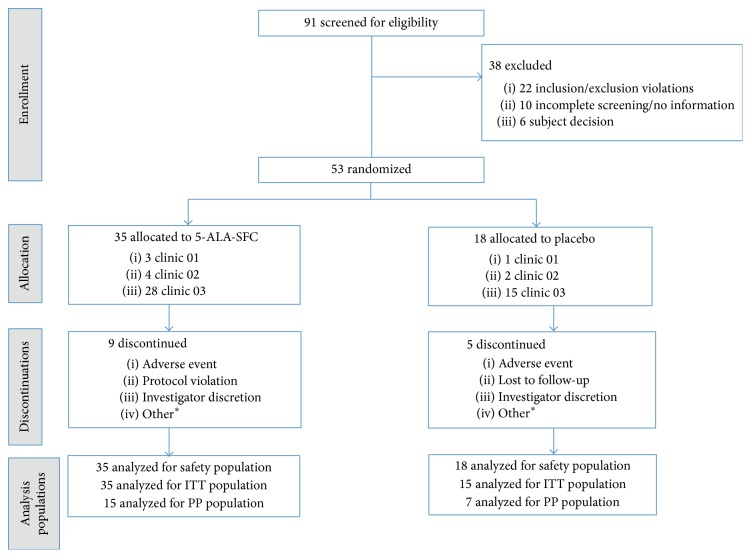
Patient disposition. ^*∗*^includes noncompliance with dosing and inability to return for visits due to work and lack of transportation.

After the run-in period, eligible patients were randomized at week 0 to either 5-ALA-SFC or matching placebo, with two patients allocated to 5-ALA-SFC for every one patient allocated to placebo. Treatment assignments were single-blinded with clinicians aware of the treatment assignment but patients unaware of the treatment they were receiving. Ninety-one patients were screened, and 53 patients were enrolled in the study (Figure 1). Patients allocated to 5-ALA-SFC received total daily dose of 100 mg 5-ALA/114.72 mg SFC beginning at week 0; 150 mg 5-ALA/172.08 mg SFC beginning at week 2; and 200 mg 5-ALA/229.42 mg SFC beginning at week 4. Total daily dose was administered in two divided doses. Prior to escalation to a higher dose, safety was assessed based on adverse reactions considered to be related to the study product and laboratory assessments, and patients unable to be escalated to the next dose level were withdrawn from the study. Patients allocated to placebo received matching capsules containing pregelatinized starch.

Patients returned at week 6 to confirm safety of the 200 mg 5-ALA/229.42 mg SFC total daily dose and then continued dosing for an additional 6 weeks. Dosing ended at week 12, and patients returned at week 14 for a follow-up safety assessment 2 weeks after the final dose of study product. Blood and urine samples for laboratory assessments were collected at each study visit.

The primary endpoint of this study was safety as assessed by incidence of adverse events. Secondary endpoints included incidence of clinically significant abnormalities in laboratory assessments and changes in plasma glucose and glycosylated hemoglobin (HbA1c).

### 2.2. Statistical Analysis

Three subject populations were defined for the purpose of analysis. The safety population included all patients who took at least one dose of study product; the intent-to-treat population (ITT) included all patients who took at least one dose of study product and had at least one postbaseline efficacy evaluation; and the per protocol population (PP) excluded patients with major protocol deviations as determined prior to database lock. Given that the study was conducted in Bahrain, the effect of fasting during the holy month of Ramadan may have impacted plasma glucose related measurements. To assess the magnitude of this impact, patients and visits affected by Ramadan were identified, and analyses of plasma glucose and HbA1c measurements were repeated excluding Ramadan impacted data.

The incidence of adverse events and incidence of clinically significant abnormalities in laboratory tests were compared using Fisher's Exact Test. In addition, shift tables from baseline were generated for all measured laboratory parameters at week 2, week 4, week 6, week 12, and week 14.

Changes from baseline in parameters of glycemic control (fasting blood glucose, HbA1c, and blood glucose 2 hours after breakfast) were compared between the two treatment groups at weeks 2, 4, 6, and 12 (HbA1c and blood glucose), weeks 8 and 10 (blood glucose only), and week 14 (HbA1c only). A general linear model was constructed with factors for treatment group, clinic, and treatment-by-clinic interaction, and baseline value as covariates. The interaction term was dropped from the model if its *p* value is greater than 0.15. Pairwise comparisons between doses of 5-ALA-SFC were tested with paired *t*-tests. The comparisons were done in pairs: week 2 (100 mg) to week 4 (150 mg), as well as week 2 (100 mg) to week 6 (200 mg), week 4 (150 mg) to week 6 (200 mg), and week 6 (200 mg) to week 12 (200 mg).

In a post hoc analysis, pairwise comparisons on HbA1c (%) mean change against baseline were tested with paired *t*-tests.

## 3. Results

Fifty-three patients (53) were enrolled at one study site, with 35 patients randomized to the 5-ALA–SFC group and 18 patients to the placebo group. Overall, the groups were comparable on baseline characteristics, with numerically higher baseline values of total cholesterol, LDL and triglycerides, and waist measurement noted in the 5-ALA-SFC group (Table 1). Antidiabetic medications taken concomitantly at baseline included drugs from the following classes: sulfonylureas, biguanides, dipeptidyl peptidase-4 inhibitors, thiazolidinediones, and combination of these drugs. At baseline, five patients (9.4%) were taking three different antidiabetic drugs, 26 (49.0%) were taking two antidiabetic drugs, and 20 patients (37.7%) were taking one antidiabetic drug at baseline. Two patients who were not taking any antidiabetic medications were withdrawn prior to week 2 visit.

There was a numerically higher percentage of patients in the 5-ALA-SFC group experiencing at least one adverse event during the study than the placebo group, but the difference was not statistically significant (45.7% 5-ALA-SFC versus 27.8% placebo; *p* = 0.206) as shown in Table 2. The most frequently reported events were gastrointestinal in nature (28.6% 5-ALA-SFC versus 16.7% placebo), which is consistent with previous experience in clinical studies with 5-ALA-SFC. Six patients in the 5-ALA–SFC group and one in the placebo group reported adverse events that resulted in premature withdrawal from the study, with three of the events in the 5-ALA-SFC group judged by the investigator to be related to the study product. A higher proportion of 5-ALA patients withdrew as a result of gastrointestinal adverse events in comparison to the placebo group. One patient in each group self-reported a mild event of hypoglycemia (2.9% 5-ALA-SFC versus 5.6% placebo), judged by the investigator to be not related to the study product. All adverse events reported were rated as mild or moderate in severity by the investigators. One serious adverse event of a nasal abscess requiring hospitalization was reported by a subject receiving 5-ALA-SFC, which was not related to study product. The patient was hospitalized for 4 days and required incision and drainage of the abscess. The event completely resolved with no sequelae. The percentage of patients who reported adverse events that were considered by the investigator as potentially related to study product was comparable in both groups at week 12 (20.07% 5-ALA-SFC versus 16.7% placebo).

**Figure 2:**
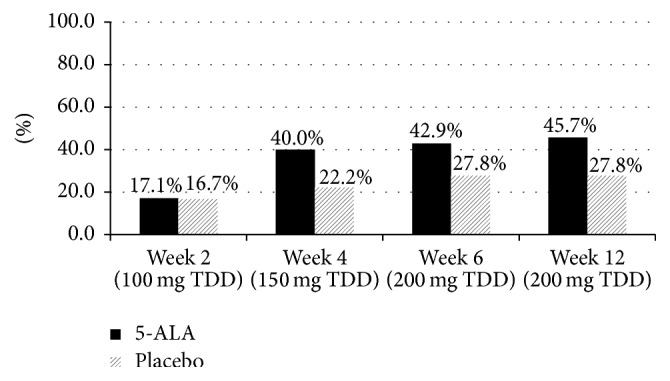
Cumulative adverse events reported at each visit.

A cumulative summary of adverse events reported during the dose escalation to 200 mg 5-ALA-SFC is presented in Table 3 and Figure 2. The number of subjects reporting at least one event remained relatively constant after week 4, with no increase in events reported upon starting the 200 mg 5-ALA dose.

Analysis of hematology, biochemistry, and urinalysis laboratory tests did not reveal any clinically significant changes from baseline during the study. Mean change from baseline for laboratory assessments of interest are shown in Table 4.

Patients in both groups experienced a decrease in weight from baseline to week 12, with a decrease of −0.2 kg in the 5-ALA group and −0.8 kg in the placebo group (*p* = 0.243).

**Figure 3:**
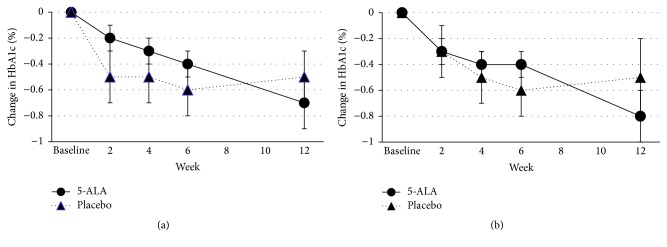
Changes in mean HbA1c during dosing period ((a) ITT population, (b) PP population).

Mean and change from baseline HbA1c levels are presented in Table 5 and Figure 3. There was no statistically significant difference in HbA1c between the two groups. A post hoc analysis showed a significant decrease in HbA1c levels from baseline in both groups in the ITT population at all visits except for week 12 placebo. In both the ITT and the PP population, patients in the 5-ALA-SFC treatment group showed a steady gradual decline in HbA1c over 12 weeks while the placebo group showed an initial decrease in HbA1c but then showed rebound after 6 weeks. At week 12, the mean HbA1c in the 5-ALA-SFC group decreased by −0.8%, compared to −0.5% in the placebo group in the PP population (Figure 3(b)).

Mean and change from baseline for fasting plasma glucose and glucose 2 hours after breakfast are presented in Table 6. Excluding glucose values from the 8 patient visits impacted by Ramadan in the study analysis resulted in no appreciable change in results; therefore, overall mean values including all assessments are presented. There was a decrease in serum glucose and glucose 2 hours after breakfast from baseline over time observed in both groups.

Post hoc additional analyses were conducted to assess the magnitude of HbA1c change from baseline for a variety of subgroups based on the class of concomitant antidiabetic medications use. HbA1c (%) change from baseline at each study visit was analyzed by class of antidiabetic drugs such as biguanides (BG), dipeptidyl peptidase-4 inhibitors (DPP-4), Sulphonylureas (SU), thiazolidinediones (TZD), and Combo (one subject taking a BG + DPP-4 and one subject taking a BG + SU). Among patients taking SU antidiabetic drugs, a gradual decline of HbA1c value was observed from week 2 through week 14 in the 5-ALA-SFC group (−0.25 (week 2), −0.37 (week 4), −0.49 (week 6), −0.95 (week 12), and −1.21 (week 14)), whereas such a trend was not seen in the placebo group (−0.64 (week 2), −0.58 (week 4), −0.75 (week 6), −0.63 (week 12), and −0.87 (week 14)). The result of analysis of HbA1c change against baseline using paired *t*-test showed a significant decrease against baseline in the 5-ALA-SFC group at week 12 in the patients taking SU class of antidiabetic drugs (5-ALA: *p* = 0.006 (ITT, *n* = 12) and *p* = 0.007 (PP, *n* = 6), placebo: *p* = 0.105 (ITT, *n* = 11) and *p* = 0.300 (PP, *n* = 5)).

## 4. Discussion

The results of the current study support that administration of the nutritional supplement 5-ALA-SFC up to doses of 200 mg 5-ALA/229.42 mg SFC per day is safe in patients with type 2 diabetes mellitus taking oral antidiabetic medications. Use of nutritional supplements is widespread in patients with diabetes [6], and assessing the safety of nutritional supplements outside of a healthy population is important to protect patient safety. Adverse events have been reported with use of nutritional supplements in healthy populations as well as in patients with comorbidities [12–14], and interactions may occur between nutritional supplements and antidiabetic drugs that could alter pharmacokinetics and therapeutic effect [6]. Oxidative stress and ROS generation are considered to induce inflammation and cause complication in DM patients [4, 5]. Thus, nutritional supplements with antioxidant properties can be beneficial in the diabetic population [4, 15]. 5-ALA-SFC has been reported to activate electron transport chain in mitochondria and further induce heme oxygenase-1, resulting in prevention of ROS production and elimination of ROS, respectively [16]. 5-ALA-SFC has been studied worldwide in patients with diabetes to ensure its safe use [7, 9, 10]. Patients enrolled in the current study took a variety of concomitant antidiabetic medications, in some patients as many as three oral agents. There were no unsafe interactions between 5-ALA and oral antidiabetic medications reported. The most frequent events reported in the study were gastrointestinal in nature, which is consistent with the known safety profile observed in prior studies among patients with diabetes mellitus. As the dose was escalated to 200 mg of 5-ALA, there was no increase in adverse events; therefore, the study supports the tolerability of this dose. Analysis of clinical laboratory test results showed no clinically significant change in liver function tests, renal function tests, or iron parameters throughout study.

Hypoglycemia could be a concern with use of a nutritional supplement in conjunction with oral antidiabetic agents, but there was no difference in hypoglycemia between patients receiving 5-ALA and placebo, with one patient self-reporting hypoglycemia in each treatment group. These results are consistent with a study conducted in Japan assessing the safety of 5-ALA-SFC in 45 patients with diabetes treated with oral antidiabetic medications, which showed doses of 15 mg and 50 mg of 5-ALA-SFC to be well tolerated by patients [7].

This pilot study also provided an opportunity for exploratory evaluation of the effect of 5-ALA-SFC on glycemic control in patients with type 2 diabetes mellitus, although it was not powered to detect differences in any of the efficacy measures. The study demonstrated that the 5-ALA group had constant gradual decrease in HbA1c over 12 weeks during study drug dosing, while the placebo group showed an initial decrease but rebounded after 6 weeks. HbA1c reflects glycemic control over the previous several months [17], and the American Diabetes Association recommends that HbA1c be assessed approximately every 3 months [18]. It would be expected that the impact of 5-ALA-SFC on glycemic control would require several months to be reflected in a change in HbA1c, and a study of longer duration and larger sample size is likely required to show a statistical difference in glycemic control as measured by HbA1c.

Daily blood glucose measurements were collected in the study by patients prior to breakfast and 2 hours after breakfast through home use of a glucometer. Results of the glucose analyses did not reveal any sustained statistically significant differences between the two treatment groups. Previous clinical studies however have shown significantly reduced blood glucose levels, using a 2-hour oral glucose tolerance test (OGTT). In a study conducted in Hawaii in 154 patients with HbA1c between 5.8 and 7.0%, significant decreases in 2-hour OGTT were seen after 12 weeks of 15 mg as well as 50 mg of 5-ALA. The reduction was greater in patients with 2-hour OGTT results at baseline ≥140 mg/kL [9]. A study conducted in Japan in 212 subjects with HbA1c between 6.1 and 7.1% or fasting plasma glucose level between 105 and 125 mg/dL also showed a decrease in fasting plasma glucose, glycoalbumin, and 2-hour OGTT after 12 weeks of 15 mg of 5-ALA [10]. Glucose serum levels in the current study were collected through patient self-monitoring during a 3-month period and reflect real-world data, as compared to a glucose challenge test in a controlled, monitored setting in the studies conducted in Hawaii and Japan, which may account for the difference in the results. Overall, the results of the current study combined with previous clinical and preclinical data support that 5-ALA could offer benefits in glycemic control in the diabetic population with minimal risk.

The changes in glycemic control noted in the placebo group during the first 6 weeks of the study suggest that blinded study drug dosing may have mediated a change in patient behaviour that improved patient compliance with diet, exercise, and/or antidiabetic medications, which could have led to this improvement in glycemic control. This finding supports the stepwise treatment recommendations beginning with lifestyle changes including dietary modifications.

Clinical research studies support the development of new therapeutic options, and country-specific studies are important to generate data in a local population. The number of research studies conducted in the Middle East is increasing, and local ethical guidelines are being implemented on a country-specific basis [19]. In Bahrain, the Ministry of Health issued the Ethical Guidelines for Health Research in 2009 [19]. Studies such as the current investigation, which has been conducted in compliance with good clinical practices, provide experience and training to local research staff thereby furthering the development of research practices, and have the potential to improve the health of the local population.

There are several limitations in the current study. The primary objective was to assess safety of escalating doses of 5 ALA-SFC; therefore, the study was not powered to detect differences in efficacy. Glucose measurements of fasting and 2 hours after breakfast were calculated from data collected via glucometer, which relies heavily on patient compliance with daily monitoring and correct operation of the device. The study population enrolled in this study had a wide range of weight (BMI (21.0–43.9 kg/m^2^)) and baseline glucose control (HbA1c: 6.6–10.2%), with the potential for variable efficacy responses across this spectrum.

The patients in the ITT population who were not in compliance with dosing and daily self-monitoring of glucose were excluded, and the PP population was formed with the rest of the patients in compliance. It seems that the PP population represents the patients most compliant to study procedures including dosing and the reliable dosing would be expected to improve HbA1c levels.

In the groups of patients taking SU antidiabetic drugs, the results of analysis calculated by paired *t*-test each week against baseline showed that HbA1c decrease from baseline was significantly different at week 12 only in the 5-ALA-SFC group (*p* = 0.006 (*n* = 12)). SU antidiabetic drugs are powerful glucose lowering agents, and it is reported that a long-term or high-dose use would undermine pancreas function and that SU drugs should not be given to patients who have kidney disorder, including the elderly. 5-ALA-SFC might help patients whose HbA1c level is not reduced in spite of taking long or high dosing of SU drug. A possible contribution of 5-ALA-SFC in treating the patients taking SU drugs can be explained by recovering of insulin sensitivity by enhancing glucose metabolism through activation of mitochondrial functions including TCA cycle activity. SU drugs are inducers of insulin secretion from pancreatic beta cells. Patients treated with SU drugs for a long period and keeping high levels of blood glucose and HbA1c were considered to be insulin resistant. Since 5-ALA-SFC promotes insulin sensitivity, a combination of SU drugs and 5-ALA-SFC is expected to be beneficial for patients taking SU dugs.

## 5. Conclusion

This study in patients with type 2 diabetes mellitus living in Bahrain supports that use of 5-ALA-SFC up to 200 mg per day taken as 2 divided doses is safe in patients taking concomitant oral antidiabetic medications. 5-ALA-SFC may offer benefits in the diabetic population, and larger studies enrolling a greater number of patients over longer periods of time are required to further define the effect of 5-ALA-SFC on glycemic control in this population.

## Figures and Tables

**Table 1:** Baseline demographics and disease characteristics.

	5-ALA-SFC		Placebo	
	*n*	Result	*n*	Result
Age (years)	35	52.4 (6.51)	15	51.9 (5.07)
Sex (male)	35	33 (94.3)	15	13 (86.7)
Fasting plasma glucose (mg/dL)	33	142.3 (21.87)	15	146.4 (31.35)
Plasma glucose 2 hrs after meal (mg/dL)	33	189.4 (47.96)	14	191.9 (57.97)
HbA1c (%)	35	7.8 (0.88)	15	8.0 (0.91)
Total cholesterol (mg/dL)	34	175.6 (36.14)	15	158.8 (31.27)
LDL (mg/dL)	34	115.4 (33.87)	15	102.0 (26.37)
HDL (mg/dL)	34	41.1 (10.80)	15	41.5 (10.25)
Triglycerides (mg/dL)	34	167.0 (89.17)	15	133.7 (55.28)
Waist (cm)	34	98.6 (21.12)	15	90.3 (17.35)
BMI (kg/m^2^)	35	30.8 (5.72)	15	29.3 (5.44)
Weight (kg)	35	89.8 (17.02)	15	86.6 (18.89)
Systolic BP (mmHg)	35	137.7 (16.67)	15	131.3 (15.88)
Diastolic BP (mmHg)	35	80.7 (10.73)	15	76.2 (8.97)
Heart rate (b/min)	24	78.4 (11.77)	9	72.1 (8.46)

All values reported as mean (SD) except sex reported as *n* (%).

**Table 2:** Summary of adverse events.

	5-ALA-SFC *n* = 35	Placebo *n* = 18
Subjects reporting at least one event^*∗*^	16 (45.7%)	5 (27.8%)
Subjects reporting at least one related event	7 (20%)	3 (16.7%)
Subjects reporting at least one severe event	0	0
Subjects reporting at least one event leading to study discontinuation	6 (17.1%)	1 (5.6%)

^*∗*^ *p* = 0.206.

**Table 3:** Cumulative summary of treatment emergent adverse events over time.

	Week 2 (100 mg 5-ALA-SFC) (*n* = 35)	Week 4 (150 mg 5-ALA-SFC) (*n* = 35)	Week 6 (200 mg 5-ALA-SFC) (*n* = 35)	Week 12 (200 mg 5-ALA-SFC) (*n* = 35)	Week 12 (Placebo) (*n* = 18)
Subjects reporting at least one event	6 (17.1%)	14 (40.0%)	15 (42.9%)	16 (45.7%)	5 (27.8%)

Abdominal distension	0	1 (2.9%)	1 (2.9%)	1 (2.9%)	0
Abdominal pain upper	1 (2.9%)	2 (5.7%)	2 (5.7%)	2 (5.7%)	0
Alopecia	0	1 (2.9%)	1 (2.9%)	1 (2.9%)	0
Blood glucose increased	0	0	1 (2.9%)	1 (2.9%)	0
Constipation	0	1 (2.9%)	1 (2.9%)	1 (2.9%)	0
Cough	1 (2.9%)	2 (5.7%)	2 (5.7%)	2 (5.7%)	0
Decreased appetite	0	1 (2.9%)	1 (2.9%)	1 (2.9%)	0
Diarrhea	2 (5.7%)	2 (5.7%)	2 (5.7%)	2 (5.7%)	2 (11.1%)
Dyspepsia	1 (2.9%)	1 (2.9%)	1 (2.9%)	1 (2.9%)	1 (5.6%)
Erectile dysfunction	0	0	1 (2.9%)	1 (2.9%)	0
Fatigue	0	1 (2.9%)	2 (5.7%)	2 (5.7%)	0
Feces discolored	0	1 (2.9%)	1 (2.9%)	1 (2.9%)	0
Feces hard	0	1 (2.9%)	1 (2.9%)	1 (2.9%)	0
Gastrointestinal disorder	0	0	1 (2.9%)	1 (2.9%)	0
Groin pain	0	0	0	0	1 (5.6%)
Headache	0	1 (2.9%)	1 (2.9%)	2 (5.7%)	1 (5.6%)
Hypoglycemia	0	0	0	1 (2.9%)	1 (5.6%)
Hypokalemia	0	0	0	0	1 (5.6%)
Nasal abscess	0	0	0	1 (2.9%)	0
Nasal congestion	1 (2.9%)	1 (2.9%)	1 (2.9%)	1 (2.9%)	0
Nausea	0	1 (2.9%)	1 (2.9%)	1 (2.9%)	0
Palpitations	0	0	1 (2.95)	1 (2.9%)	0
Pyrexia	0	1 (2.9%)	1 (2.9%)	1 (2.9%)	0

**Table 4:** Laboratory parameters at week 6 and week 12.

	5-ALA-SFC			Placebo		
	*n*	Mean	Change from baseline^a^ Mean (SE)	*n*	Mean	Change from baseline^a^ Mean (SE)
Total cholesterol (mg/dL)						
Week 6	27	166.9	−5.2 (3.7)	13	150.3	−7.6 (5.5)
Week 12	25	181.4	6.8 (3.5)	13	160.2	−0.1 (5.0)

LDL cholesterol (mg/dL)						
Week 6	27	109.4	−2.9 (3.2)	13	92.4	−11.8 (4.7)
Week 12	25	121.4	7.6 (3.3)	13	101.2	−4.0 (4.6)

HDL cholesterol (mg/dL)						
Week 6	27	40.2	−0.8 (1.0)	13	37.0	−1.6 (1.4)
Week 12	25	41.9	0.5 (1.0)	13	37.4	−1.1 (1.4)

Triglycerides (mg/dL)						
Week 6	27	156.6	−1.3 (14.6)	13	150.4	4.5 (21.1)
Week 12	25	163.9	3.2 (11.1)	13	159.0	11.9 (15.4)

BUN (mg/dL)						
Week 6	25	14.05	1.19 (0.573)	11	13.42	−0.43 (0.665)
Week 12	26	13.87	1.28 (0.606)	12	13.00	−0.54 (0.816)

Serum creatinine (mg/dL)						
Week 6	28	0.96	0.01 (0.021)	13	1.04	−0.01 (0.023)
Week 12	26	0.96	−0.00 (0.016)	13	1.06	0.01 (0.026)

Ferritin (mg/dL)						
Week 6	26	169.03	31.12 (9.480)	12	136.48	−12.59 (9.033)
Week 12	22	148.71	16.64 (11.289)	12	137.54	−11.53 (14.456)

Serum iron (mg/dL)						
Week 6	27	18.16	0.87 (1.330)	12	16.14	−2.27 (1.502)
Week 12	22	17.73	1.41 (1.409)	12	16.74	−1.67 (1.083)

Transferrin saturation (%)						
Week 6	25	31.38	3.77 (2.960)	12	28.48	−3.43 (2.562)
Week 12	21	29.93	3.37 (2.638)	12	29.55	−2.36 (1.753)

^a^Compared to baseline mean for only the subjects with a result for the visit.

**Table 5:** Change in mean HbA1c during dosing period (ITT and PP populations).

	5-ALA-SFC			Placebo		
	*n*	Mean	Change from baseline^a^ Mean (SE)	*n*	Mean	Change from baseline^a^ Mean (SE)
Intent-to-treat population						
Week 2	32	7.6	−0.2^*∗*^ (0.1)	13	7.4	−0.5^*∗*^ (0.2)
Week 4	30	7.5	−0.3^*∗*^ (0.1)	13	7.3	−0.5^*∗*^ (0.2)
Week 6	28	7.3	−0.4^*∗*^ (0.1)	13	7.2	−0.6^*∗*^ (0.2)
Week 12	25	7.1	−0.7^*∗*^ (0.2)	13	7.3	−0.5 (0.2)

Per protocol population						
Week 2	15	7.3	−0.3^*∗*^ (0.1)	6	7.6	−0.3 (0.2)
Week 4	15	7.1	−0.4^*∗*^ (0.1)	7	7.3	−0.5 (0.2)
Week 6	14	7.1	−0.4^*∗*^ (0.1)	7	7.2	−0.6 (0.2)
Week 12	14	6.8	−0.8^*∗*^ (0.2)	7	7.3	−0.5 (0.3)

^*∗*^ *p* < 0.05 compared to baseline.

^a^Compared to baseline mean for only the subjects with a result for the visit.

**Table 6:** Mean and change from baseline in fasting plasma glucose and plasma glucose 2 hours after breakfast (ITT population).

	5-ALA-SFC			Placebo		
	*n*	Mean	Change from baseline^a^ Mean (SE)	*n*	Mean	Change from baseline^a^ Mean (SE)
Fasting plasma glucose (mg/dL)						
Week 2	33	145.1	2.3 (3.4)	14	138.4	−7.3 (5.2)
Week 4	30	142.1	−0.2 (4.9)	14	145.6	−0.8 (7.2)
Week 6	27	138.0	−4.8 (3.2)	13	132.4	−9.2 (4.7)
Week 8	22	135.8	−4.5 (4.0)	12	142.7	−1.0 (5.5)
Week 10	23	140.3	−0.9 (5.6)	12	143.1	−0.9 (7.8)
Week 12	22	139.3	−3.0 (4.4)	12	139.5	−4.2 (5.9)

Plasma glucose 2 hours after meal (mg/dL)						
Week 2^*∗*^	33	189.6	−0.2 (5.1)	14	166.3	−26.5 (7.8)
Week 4	30	175.7	−12.9 (6.1)	12	173.9	−18.8 (9.9)
Week 6	27	173.4	−14.5 (5.4)	13	158.5	−27.4 (7.8)
Week 8	20	176.2	−5.6 (5.6)	11	155.0	−30.5 (11.2)
Week 10	20	175.1	−10.3 (10.5)	12	181.2	−7.6 (13.5)
Week 12	21	175.0	−8.5 (9.8)	10	154.3	−33.0 (14.2)

^*∗*^ *p* < 0.05 between groups.

^a^Compared to baseline mean for only the subjects with a result for the visit.

## Abbreviations

5-ALA: 5-Aminolevulinic acid
SFC: Sodium ferrous citrate
T2DM: Type 2 diabetes mellitus
ROS: Reactive oxygen species
BMI: Body mass index
BP: Blood pressure
BUN: Blood urea nitrogen
eGFR: Estimated glomerular filtration rate
HbA1c: Glycohemoglobin A1c
ITT: Intent-to-treat population
PP: Per protocol population
LDL: Low density lipoprotein
OGTT: Oral glucose tolerance test.
